# Exploring HMMR as a therapeutic frontier in breast cancer treatment, its interaction with various cell cycle genes, and targeting its overexpression through specific inhibitors

**DOI:** 10.3389/fphar.2024.1361424

**Published:** 2024-03-21

**Authors:** Aisha Shabir, Hina Qayoom, Burhan Ul Haq, Adel Abo Mansoor, Adil Abdelrahim, Irshad Ahmad, Abdullah Almilabairy, Fuzail Ahmad, Manzoor Ahmad Mir

**Affiliations:** ^1^ Department of Bioresources, School of Biological Sciences, University of Kashmir, Srinagar, India; ^2^ Department of Clinical Laboratory Sciences, College of Applied Medical Sciences, King Khalid University, Abha, Saudi Arabia; ^3^ Department of Medical Rehabilitation Sciences, College of Applied Medical Sciences, King Khalid University, Abha, Saudi Arabia; ^4^ Department of Family and Community Medicine, Faculty of Medicine, Al Baha University, Abha, Saudi Arabia; ^5^ Respiratory Care Department, College of Applied Sciences Almaarefa University, Diriya, Riyadh, Saudi Arabia

**Keywords:** HMMR, breast cancer, biomarkers, Cdk1, AURKA, Tpx2, mTOR, MD simulation

## Abstract

Among women, breast carcinoma is one of the most complex cancers, with one of the highest death rates worldwide. There have been significant improvements in treatment methods, but its early detection still remains an issue to be resolved. This study explores the multifaceted function of hyaluronan-mediated motility receptor (HMMR) in breast cancer progression. HMMR’s association with key cell cycle regulators (AURKA, TPX2, and CDK1) underscores its pivotal role in cancer initiation and advancement. HMMR’s involvement in microtubule assembly and cellular interactions, both extracellularly and intracellularly, provides critical insights into its contribution to cancer cell processes. Elevated HMMR expression triggered by inflammatory signals correlates with unfavorable prognosis in breast cancer and various other malignancies. Therefore, recognizing HMMR as a promising therapeutic target, the study validates the overexpression of HMMR in breast cancer and various pan cancers and its correlation with certain proteins such as AURKA, TPX2, and CDK1 through online databases. Furthermore, the pathways associated with HMMR were explored using pathway enrichment analysis, such as Gene Ontology, offering a foundation for the development of effective strategies in breast cancer treatment. The study further highlights compounds capable of inhibiting certain pathways, which, in turn, would inhibit the upregulation of HMMR in breast cancer. The results were further validated via MD simulations in addition to molecular docking to explore protein–protein/ligand interaction. Consequently, these findings imply that HMMR could play a pivotal role as a crucial oncogenic regulator, highlighting its potential as a promising target for the therapeutic intervention of breast carcinoma.

## Graphical Abstract

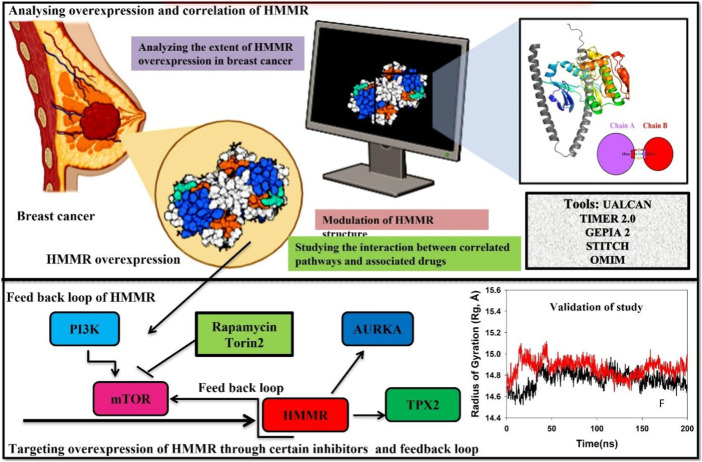

## Highlights

• The glycoprotein hyaluronan-mediated motility receptor (HMMR), encoded by the HMMR gene on chromosome 5, exhibits a spiral structure and serves diverse functions in cell growth, cancer metastasis, cellular pluripotency, and resistance to cancer treatments.
• Although HMMR expression is carefully regulated in healthy tissues, it undergoes upregulation in proliferative tissues, contributing to the invasive nature and metastasis observed in various carcinomas, including breast, colorectal, and prostate carcinomas.
• HMMR is associated with the cell cycle, particularly in the S/G2 and G2/M phases, by potentially impacting CDK1 and AURKA levels and also the pivotal PI3K/AKT/mTOR pathway that is essential for cell division. It plays a significant role in the G2/M phase by directly phosphorylating CDK1 activators and inhibitors. Due to persistent HMMR overexpression in cancer, it emerges as a promising therapeutic target. Inhibiting HMMR expression through compounds targeting key signaling pathways, such as mTOR, presents innovative therapeutic avenues, particularly in breast cancer.
• Our study underscores the multifaceted involvement of HMMR in carcinoma initiation and advancement, emphasizing its importance as a therapeutic target in breast cancer. Our study proposes specific inhibitors for further investigation in breast cancer treatment.

## 1 Introduction

Despite extensive exploration in both basic and clinical research, along with trials of potential innovative treatments, cancer persists as a significant global health challenge (Siegel et al., 2023). As per GLOBOCAN (2020) data, breast cancer continues to be one of the most frequently occurring cancers. Manifesting as a diverse malignancy, it constitutes as the foremost factor in cancer-related fatalities among women (Stickeler, 2011). The detection of molecular biomarkers that can function as prognostic and predictive markers has aided healthcare professionals in therapeutic choices. This allows for the application of a more individualized approach to treatment, optimizing therapy, and averting the scenarios of excessive treatment, insufficient treatment, and inaccurate treatment (Stickeler, 2011; Mir et al., 2023). Hyaluronan-mediated motility receptor (HMMR), also known as CD168 or RHAMM (receptor for hyaluronic acid-mediated motility), is a glycoprotein with spiral configuration encoded by the HMMR gene situated on the human chromosome 5 (5q33.2-qter) (Tolg et al., 2010). As per the available information, HMMR displays a significant coiled-coil (CC) structure containing numerous binding sites for its associates (Hardwick et al., 1992; B; Yang et al., 1994). In the beginning, HMMR was identified as an innovative hyaluronan-mediated mobility receptor and a microtubule-associated spindle assembly factor (Hofmann et al., 1998). However, extensive research now suggests that HMMR performs multiple functional roles in overseeing cell growth, the spread of cancer to other parts of the body (D. Yang et al., 2021), the preservation of cellular pluripotency (Tilghman et al., 2014), and resistance to cancer treatment (Sofi et al., 2023; H; Zhang et al., 2019) in various malignancies, such as lung carcinoma (Li W. et al., 2020), hepatic neoplasia (D. Zhang et al., 2020), urothelial carcinoma (D. Yang et al., 2021), and stomach neoplasia (Kang et al., 2020). Studies have revealed that the regulation of HMMR expression is closely managed in healthy tissues, yet it undergoes upregulation in proliferative tissues. This contributes to invasiveness and metastasis, leading to an unfavorable prognosis in various human carcinomas, including colorectal (Zlobec et al., 2008), breast (Assmann et al., 2001), and prostate carcinomas (Gust et al., 2009).

HMMR exhibits diverse functions, which vary based on whether it is located extracellularly or intracellularly. In the extracellular capacity, HMMR functions as extensively characterized receptors for hyaluronic acid (HA), regulating cell migration induced by HA, a pivotal aspect of both inflammation and the recuperative process during wound healing associates (Hardwick et al., 1992; B; Yang et al., 1994). In the intracellular capacity, it serves as a protein associated with the cell cycle, overseeing the formation of the mitotic spindle and microtubules (Assmann et al., 2001). Significantly, these inherent roles of HMMR are frequently disrupted in cancer, leading to growth benefits and advancements in the disease (Y.-T. Chen et al., 2018). HMMR expression in most healthy tissues at a balanced state is generally minimal, and its expression is triggered in response to inflammatory signals (Tolg et al., 2021). On the other hand, human neoplasms have been reported to display increased concentrations of HMMR, and this upregulation is often associated with metastatic tendencies, formidable traits, and an adverse prognosis in instances of prostate and hematological carcinomas (Y.-T. Chen et al., 2018). The excessive expression of HMMR also operates as a standalone predictor of prognosis in many cancer types (Mele et al., 2017; Song et al., 2018).

HMMR shows potential to indirectly aid in the microtubule assembly by facilitating the precise positioning of TPX2, thereby promoting the efficient activation of aurora kinase at specific centers crucial for microtubule formation (H. Chen et al., 2014). HMMR serves as a collaborative protein with TPX2. The trilateral association of these two proteins, along with dynein, upholds the structural integrity of the spindle and enhances the concentration of spindle poles (Maxwell et al., 2003). TPX2 acts as a co-activator of AURKA and plays a dual role: it alone can boost kinase activity beyond its baseline levels and is indispensable for achieving optimal kinase functionality. Given that approximately 40%–60% of TPX2 forms a complex with HMMR during mitosis in human cells, HMMR emerges as a significant contributor of the cellular processes. HMMR does not possess a transmembrane domain, allowing it to associate with various receptors embedded in the membrane, such as CD44, and also transforming growth factor β1 (TGF-β1) (Bayliss et al., 2003). This interaction triggers the intracellular signaling pathway related to cellular migration. A mechanism by which HMMR might enhance cellular multiplication is by amplifying the function of M-phase-encouraging factor CDK1 activity through the fortification of Cdc2 mRNA levels in a hyaluronic acid-dependent manner (Mohapatra et al., 1996). The CDK1/cyclin B complex governs the initiation of mitosis by phosphorylating diverse spindle-related proteins, such as importin, to regulate the efficient formation of microtubules and segregation of chromosomes (Guo et al., 2019). Consequently, the overexpression of HMMR may expedite the premature initiation of mitosis, resulting in a genetic modification that supports the growth of tumors through an upsurge in CDK1 activity.

Cell cycle advancement via the S/G2 and G2/M phases in both meiosis and mitosis relies on the vital activation of Cdk1, a catalytic unit of the M phase-promoting factor (MPF). During the G2/M phase, the mTOR pathway assumes a crucial role in facilitating cell division by directly phosphorylating the activators and inhibitors of CDK1. This underscores the importance of AKT in regulating the cell cycle and highlights the importance of the PI3K/AKT pathway in promoting cellular division. Due to the persistent overexpression of HMMR in cancer and its manifold functions in cancer initiation and advancement, it holds substantial promise as a target for cancer treatment. Consequently, the identification of substances that obstruct HMMR expression in tumors may present innovative therapeutic approaches to curb tumor growth. Here, we demonstrate HMMR and its correlation with certain potential genes such as *AURKA*, *TPX2*, and *CDK1* that are involved in the cell cycle and are useful for the activation of certain pathways, which may be targeted to reduce HMMR overexpression. We have further tried to demonstrate how HMMR can be inhibited through certain compounds that are key regulators of important signaling pathways which aid in breast cancer progression. It was seen through different databases that HMMR was highly upregulated in various malignancies, including breast cancer, and HMMR has a correlation with AURKA, TPX2, and CDK1, which are also involved in the upregulation of mTOR signaling pathways. Therefore, in this study, we propose that targeting mTOR through certain inhibitors like rapamycin and Torin2 is of great significance as targeting the mTOR pathway will inhibit HMMR overexpression due to a strong correlation between HMMR and the genes involved in mTOR and the cell cycle pathway (*AURKA*, *TPX2*, and *CDK1*). Hence, HMMR holds a great therapeutic significance.

## 2 Methodology

### 2.1 Predicting a potential target gene associated with the respective disease (breast cancer)

The Gene Cards Database (https://www.genecards.org/) (Safran et al., 2010), OMIM (https://www.omim.org/) (Hamosh et al., 2000), and STITCH (https://www.omim.org/) (Kuhn et al., 2010) were utilized to retrieve extensive and user-friendly details regarding genes that are either anticipated or established as potential biomarkers linked with breast cancer. These amalgamated databases were utilized to collect information on diverse genes associated with breast cancer. The term “breast cancer” was applied as a primary keyword in this investigation.

### 2.2 UALCAN

UALCAN serves as a comprehensive online data repository for the exploration of cancer omics data (Chandrashekar et al., 2017). Developed using PERL-CGI, it offers high-quality graphs and box plots, facilitating convenient access to publicly accessible cancer OMICS data from sources such as TCGA, CPTAC, and CBTTC. The platform aids in the identification of potential biomarkers for the *in silico* validation of selected genes, and it provides comprehensive expression profiles for these genes across various carcinomas. Moreover, UALCAN offers valuable patient survival information for protein-coding genes, as well as miRNAs and lncRNAs. To investigate the impact of HMMR expression on various cancer types, data from the UALCAN dataset were consulted.

### 2.3 TIMER 2.0

A thorough tool for methodologically analyzing the expression and estimate of immunological filtrates across various carcinomas is TIMER 2.0 (http://timer.comp-genomics.org/) (Li T. et al., 2020). Methods like CIBERSORT, quanTIseq, MCPcounter, and EPIC have made it possible to observe the correlation between immune cell types, gene expression, and mutation status in the database. To gain insights into the gene expression patterns of HMMR, a heat map of the protein was created using TIMER 2.0 across all malignancies.

### 2.4 Constructing a shared network that connects the disease biomarkers

A search of the STRING database (https://stringdb.org/) (Szklarczyk et al., 2021) may be used to identify important regulatory genes implicated in the illness and look for information on protein–protein interactions (PPIs) (Lehne and Schlitt, 2009). It consists of a plethora of data regarding known and predicted protein–protein interactions of several species (Szklarczyk et al., 2016). PPIs were identified using STRING, with a minimum confidence level of >0.4 as the cutoff value. Only “*Homo sapiens*” was included in the inquiry, and high-confidence ratings of more than 0.7 were not shared with STRING prior to the verified targets being sent there. In the end, PPI data were located again. The primary targets for treating breast cancer were determined to be the top proteins with the greatest levels of expression.

### 2.5 bc-GenExMiner

The annotated BC transcriptomic and RNA-Seq data have been made accessible online via the Breast Cancer Gene-Expression Miner V4.5 (http://bcgenex.ico.unicancer.fr/) (!!! INVALID CITATION !!! (Jézéquel et al., 2012; Jezequel et al., 2013)). It was applied to investigate the relationship between HMMR expression levels and certain clinical characteristics of individuals with breast tumors. The findings of the HMMR expression investigation were examined in connection with several target genes.

### 2.6 GEPIA2

Using a common processing pipeline, GEPIA2, a comprehensive online tool (http://gepia2.cancer-pku.cn/), analyzes the RNA sequencing expression data on 9,736 tumors and 8,587 normal samples from the TCGA and GTEx projects. Customizable services include differential expression analysis of tumors vs. normal, profiling based on the pathological stages or cancer kinds, studies of patient survival, identification of comparable genes, correlation analysis, and dimensionality reduction analysis. RNA-seq datasets generated by a standard procedure from the UCSC Xena project were utilized by GEPIA2 (Tang et al., 2019). Using the GEPIA2 portal, the HMMR expression in pan carcinomas was evaluated. We also generated a heat map showing the patterns of HMMR expression in several TCGA tumors.

### 2.7 Analyzing networks involving active genes and the interactions of disease target genes within the pathways

Utilizing Cytoscape 3.8.0 (http://cytoscape.org/.ver.3.8.0), we established an intricate network of interactions involving specific genes associated with the cell cycle, a target gene implicated in malignancy, and a gene pathway. Cytoscape (Kohl et al., 2011), an open-source software platform, brings about the visualization of biological pathways and interactions of molecular networks. Moreover, these networks can be integrated with annotations, diverse data types, and gene expression profiles for comprehensive analysis using Cytoscape.

### 2.8 Modelling of proteins and protein–protein docking

Molecular modeling of HMMR was conducted through a threading approach in AlphaFold, Google Colab. The resultant best model underwent additional refinement processes, addressing steric clashes and incorporating hydrogen and missing atoms. This refinement was executed using the Galaxy server (https://galaxy.seoklab.org/). All the refined models underwent comprehensive quality analysis using SAVES v6.0, and their secondary structure information was extracted from the Ramachandran plot. Subsequent to the quality assessment, protein–protein docking of the modeled proteins aimed at understanding the interactions between HMMR, AURKA, and CDK1. This docking procedure was performed utilizing the HDOCK web server. Post-analysis of protein–protein interactions was carried out through Maestro (Schrodinger LLC., United States).

### 2.9 Molecular docking

The exploration of molecular docking involved the target proteins HMMR and the ligands rapamycin and Torin2. AutoDock version v 4.2.6 (https://autodock.scripps.edu/) was utilized for these investigations. Each docking analysis consisted of three iterations, resulting in a total of 50 solutions for each case. The specified parameters included 2,500,000 evaluations, a population size of 500, and a maximum of 27,000 generations, while the remaining factors were maintained at the default values. Following the docking procedure, RMSD collection maps were generated by re-clustering at tolerance levels of 0.5 Å, 1 Å, and 2 Å. This iterative process aimed to pinpoint the optimal cluster characterized by a substantial population count and the lowest score of energy.

### 2.10 Molecular dynamics simulation

Molecular dynamics (MD) simulations were carried out on complexes involving HMMR with rapamycin, HMMR with Torin2, HMMR with AURKA, and HMMR with CDK1 utilizing Desmond 2020.1 by Schrödinger, LLC. The OPLS-2005 force field and an explicit solvent model incorporating TIP3P water molecules enclosed within a recurrent boundary salvation box (10 Å × 10 Å x 10 Å) were employed (Jorgensen et al., 1983). To balance the charge at 0.15 M, Na + ions were introduced, and the NaCl solution was added to mimic a biological environment. The system underwent equilibration using an NVT ensemble for 10 ns to stabilize the protein–ligand complexes. Subsequently, a brief equilibration and minimization phase was conducted using an NPT ensemble for 12 ns. The NPT ensemble utilized the Nose–Hoover chain coupling scheme for temperature fluctuation with a relaxation time of 1.0 ps, and pressure was maintained at 1 bar throughout every simulation. Employing a time step of 2 fs, the Martyna–Tuckerman–Klein chain coupling scheme was employed for pressure control with a relaxation time of 2 ps. The method that calculated long-range electrostatic interactions with a fixed radius for Coulomb interactions set at 9 Å was the particle-mesh Ewald method. Utilizing a RESPA integrator which had a time step of 2 fs for each trajectory, the final production run extended for 200 ns each. To evaluate the consistency of the MD simulations, various parameters, including root-mean-square deviation (RMSD), root-mean-square fluctuation (RMSF), the radius of gyration (Rg), and the number of hydrogen bonds, were computed (Martyna et al., 1992; Martyna et al., 1994; Toukmaji and Board Jr, 1996).

### 2.11 Binding free energy analysis

The determination of binding free energies for ligand–protein complexes involved employing the generalized Born surface area (MM-GBSA) approach in conjunction with molecular mechanics. The Prime MM-GBSA binding free energy was computed using the thermal mmgbsa.py Python script, which accessed the simulation trajectory for the last 50 frames with a 1-step sampling size. The calculation of Prime MM-GBSA binding free energy (in kcal/mol) adhered to the principle of additivity. This entailed summing up individual energy components, encompassing covalent, hydrogen bond, van der Waals, columbic, self-contact, lipophilic, and solvation energies of both the protein and the ligand. The formula employed for computing ΔG_bind_ is outlined as follows:

$${∆G}_{bind}={\Delta G}_{MM}+{\Delta G}_{Solv}-{\Delta G}_{SA}.$$

Here,

- ∆ G_bind_ depicts the binding free energy.
- ∆ G_MM_ depicts the difference between the free energies of ligand–protein complexes and the total energies of the protein and ligand in the isolated form.
- ∆ G_Solv_ represents the difference in the GSA solvation energies of the ligand–receptor complex and the sum of the solvation energies of the receptor and the ligand in the unbound state.
- ∆ G_SA_ signifies the difference in the surface area energies of the protein and the ligand.

## 3 Results

### 3.1 Overexpression and upregulation of HMMR across various cancer types

**FIGURE 1:**
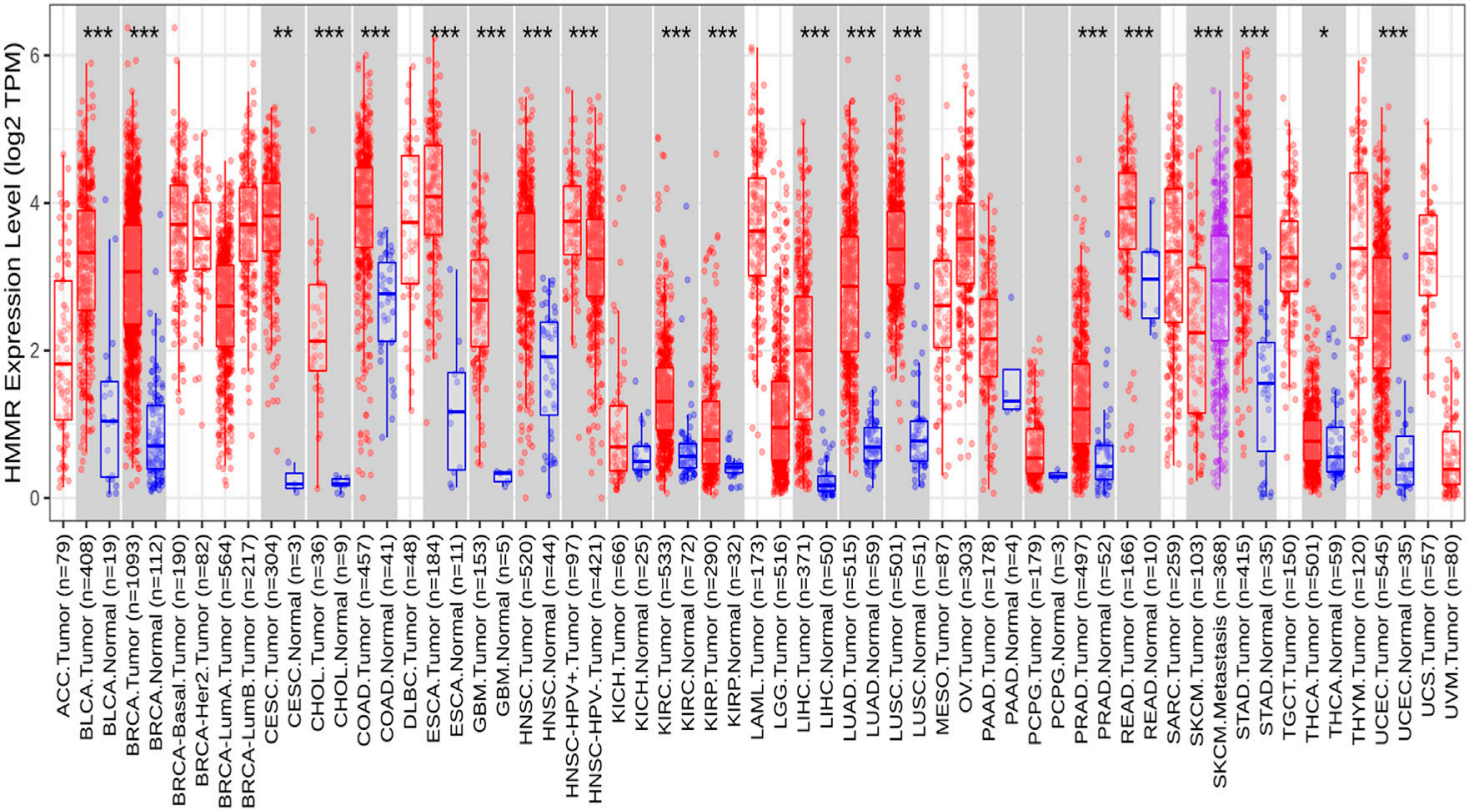
Differential expression of HMMR within carcinoma and adjacent normal tissues across all TCGA tumors from the TIMER 2.0 database. The box plots demonstrate that HMMR is highly upregulated in several malignancies. The statistical significance was computed by the Wilcoxon test and is annotated by the number of stars (*p-value<0.05; **p-value<0.01; ***p-value<0.001).

**FIGURE 2:**
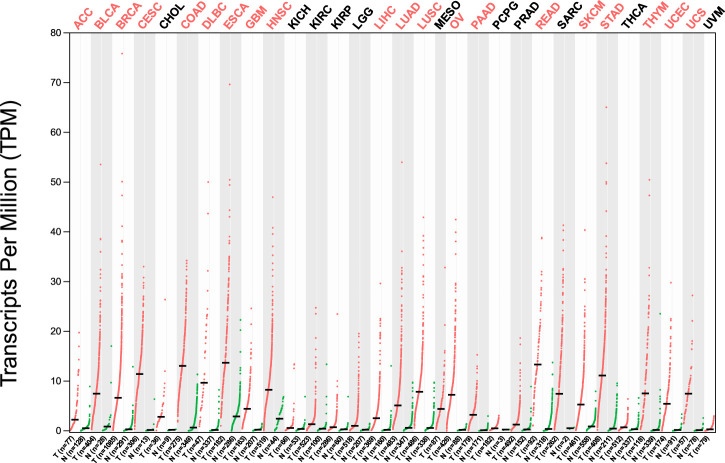
Differential gene expression of HMMR between cancerous and adjacent normal tissues across all TCGA tumors from the GEPIA 2 database. The heat map demonstrates that HMMR is highly overexpressed in several malignancies.

**FIGURE 3:**
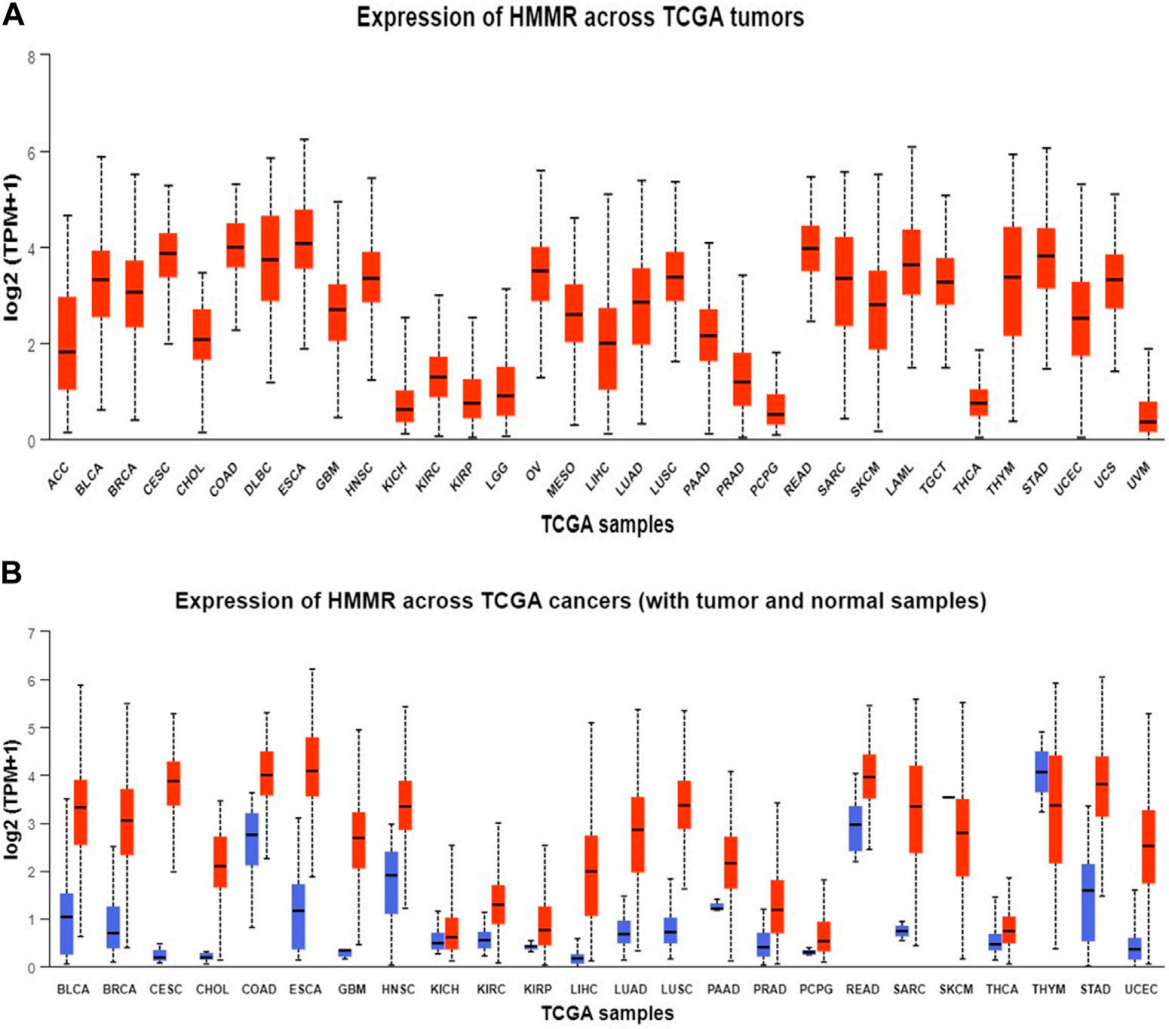
Analysis of mRNA level expression; **(A)** Expression of HMMR across TCGA tumours **(B)** Expression of HMMR across TCGA cancers (with tumour and normal samples).

**FIGURE 4:**
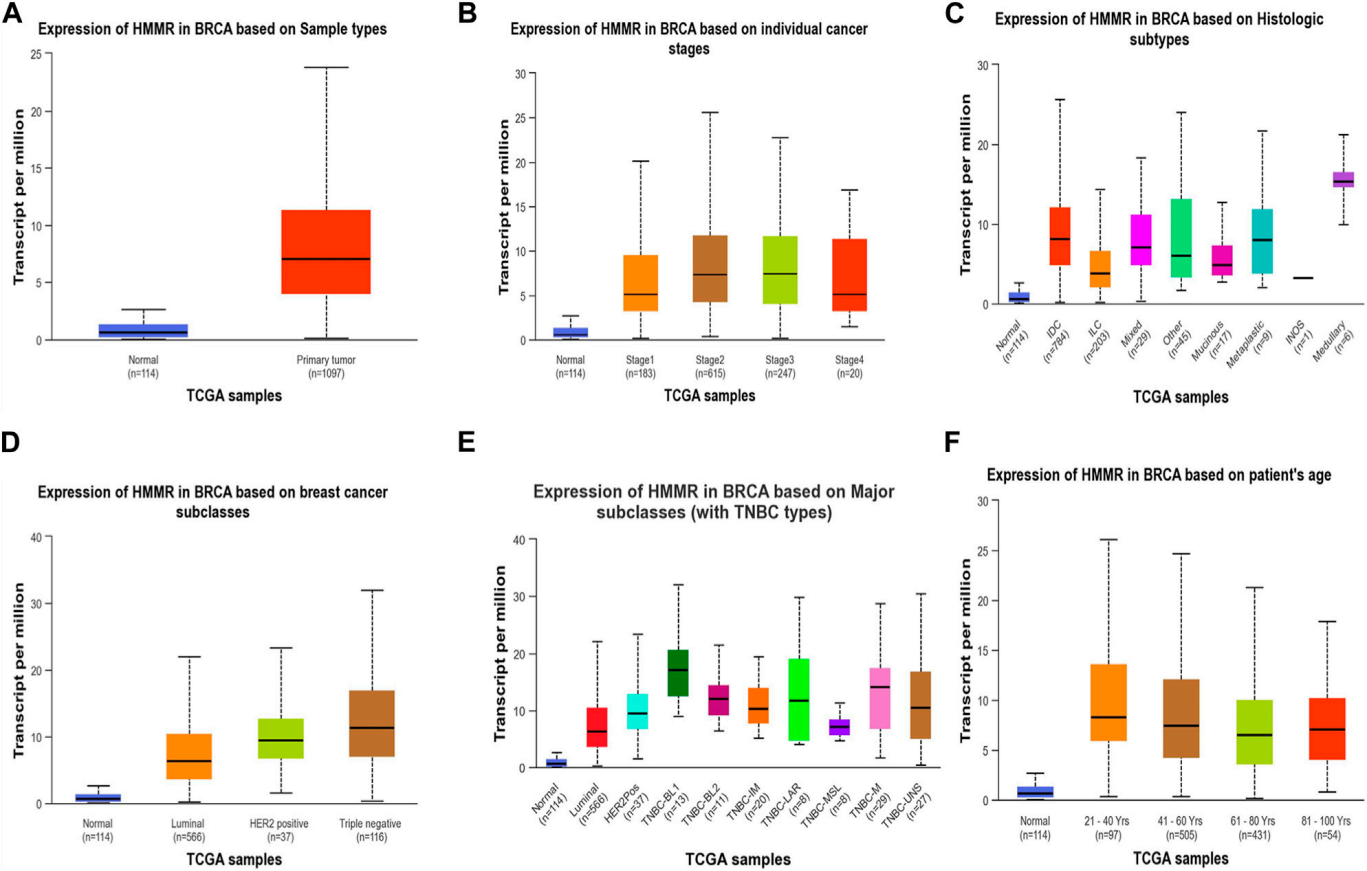
Analysis of HMMR on the basis of **(A)** sample types**, (B)** individual cancer stages, **(C)** histologic subtypes**, (D)** breast cancer sub-classes, **(E)** major subclasses, and **(F)** patient age. The bar graphs depict the elevated levels of HMMR at all levels in breast cancer.

The analysis of HMMR expression patterns was examined through The Cancer Genome Atlas (TCGA) datasets utilizing TIMER 2.0 and GEPIA2 analyses. The research revealed increased HMMR expression across numerous malignancies, as depicted in the heat map (Figures 1, 2). Furthermore, the level of mRNA for HMMR was scrutinized using the UALCAN database by comparing TCGA tumor samples with their corresponding normal samples (Figures 3, 4). It was revealed that HMMR was highly upregulated in all carcinomas, including ESCA, COAD, CESC, and READ, followed by BRCA and STAD.

The investigation unveiled an upregulation and overexpression of HMMR across almost all tumor samples, including various breast cancer stages, such as individual cancer stages, histological subtypes, breast cancer sub-classes, TNBC subtypes, and those based on patient age (Figure 4). All these databases represented the significant overexpression of HMMR in breast cancer at all stages and levels, as compared to normal samples and its involvement and importance as a therapeutic target.

### 3.2 Protein–protein interaction through STRING and Cytoscape

**FIGURE 5:**
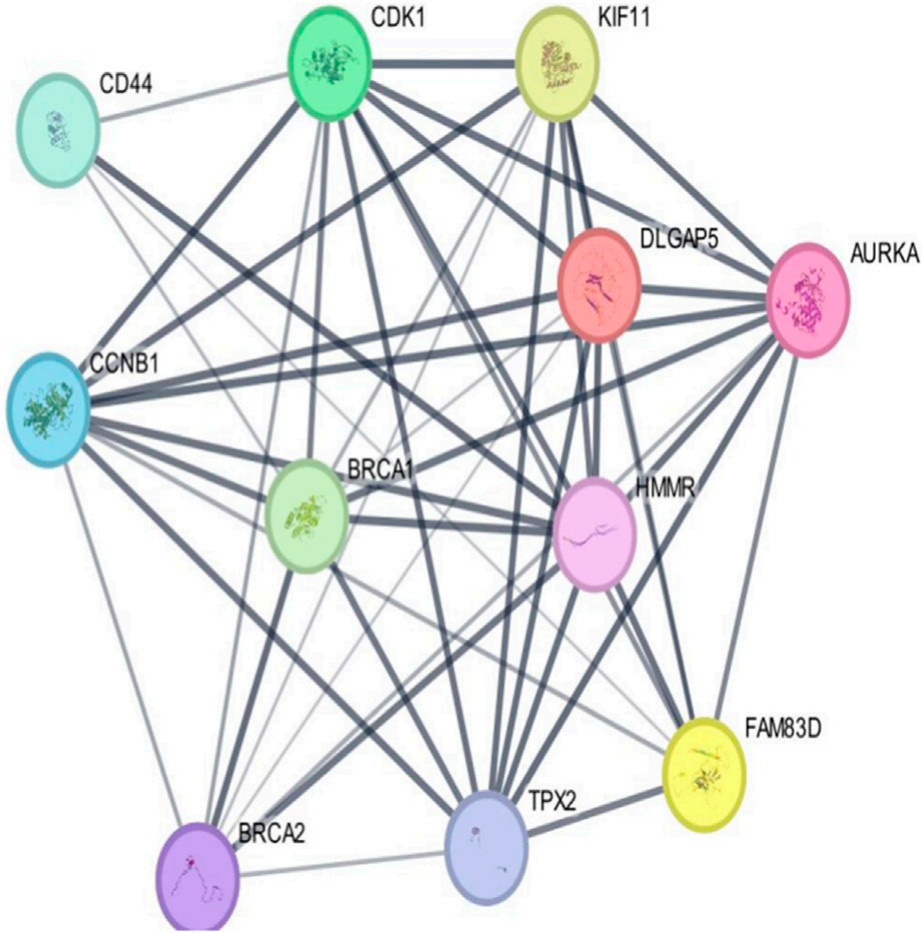
Outcomes arising from the interplay among interconnected networks of shared target genes. The protein–protein interaction (PPI) network representing these common target genes is presented, with nodes representing the target genes and their relationships visualized through edges. This network effectively communicates the connections between the target genes. The nodes are color-coded; cyan and purple denote confirmed interactions; green, blue, and purple indicate anticipated interactions; and yellow, sky blue, and light green represent other interactions. The combination of node colors and their spatial arrangement provides insights into the three-dimensional configuration of the target genes.

As per the PPI diagram depicted in Figure 5, examining the shared target genes reveals a network consisting of 11 nodes and 47 edges. Furthermore, to construct a PPI network based on the amalgamated network targets, PPI data from the STRING platform were central proteins within the network. These findings imply that the chosen components have a strong affinity, making them promising gene targets for addressing breast carcinoma. The data were incorporated into the Cytoscape application. Notably, target genes such as AURKA, CDK1 and TPX2 exhibited an elevated frequency of protein interactions suggesting their potential role as central proteins within the network. These findings imply that the chosen components have a strong affinity, making them promising gene targets for addressing breast carcinoma.

### 3.3 Pathway enrichment analysis and Gene Ontology

**FIGURE 6:**
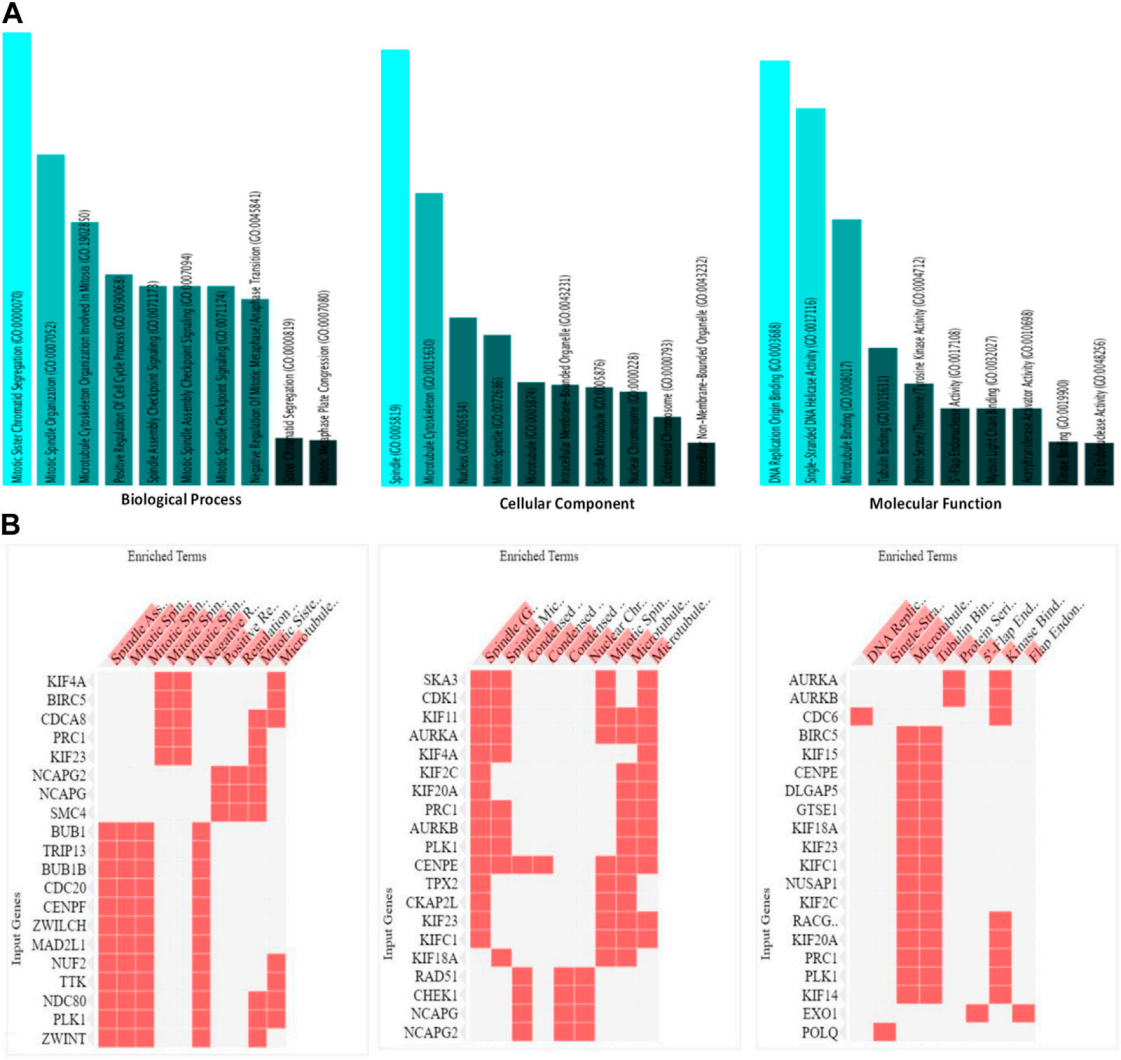
**(A)**. Analysis of the processes of utmost significance based on the numbers of associated target genes and the outcomes of the Gene Ontology (GO) categories. **(B)**. Heat map representation of the involvement of HMMR with other genes in biological, cellular, and molecular processes.

An exploration of Gene Ontology for the shared target genes indicated a primary focus on biological processes related to mitotic sister chromatid segregation and mitotic spindle organization. In terms of cellular components, the emphasis was on spindle and microtubule cytoskeleton. Molecular functions included DNA replication origin binding, single-stranded DNA helicase activity, and microtubule binding (Figure 6). These findings underscore a distinct involvement of HMMR in the cell cycle, encompassing multiple pathways and intricate interactions among them.

### 3.4 Gene correlation analysis

**FIGURE 7:**
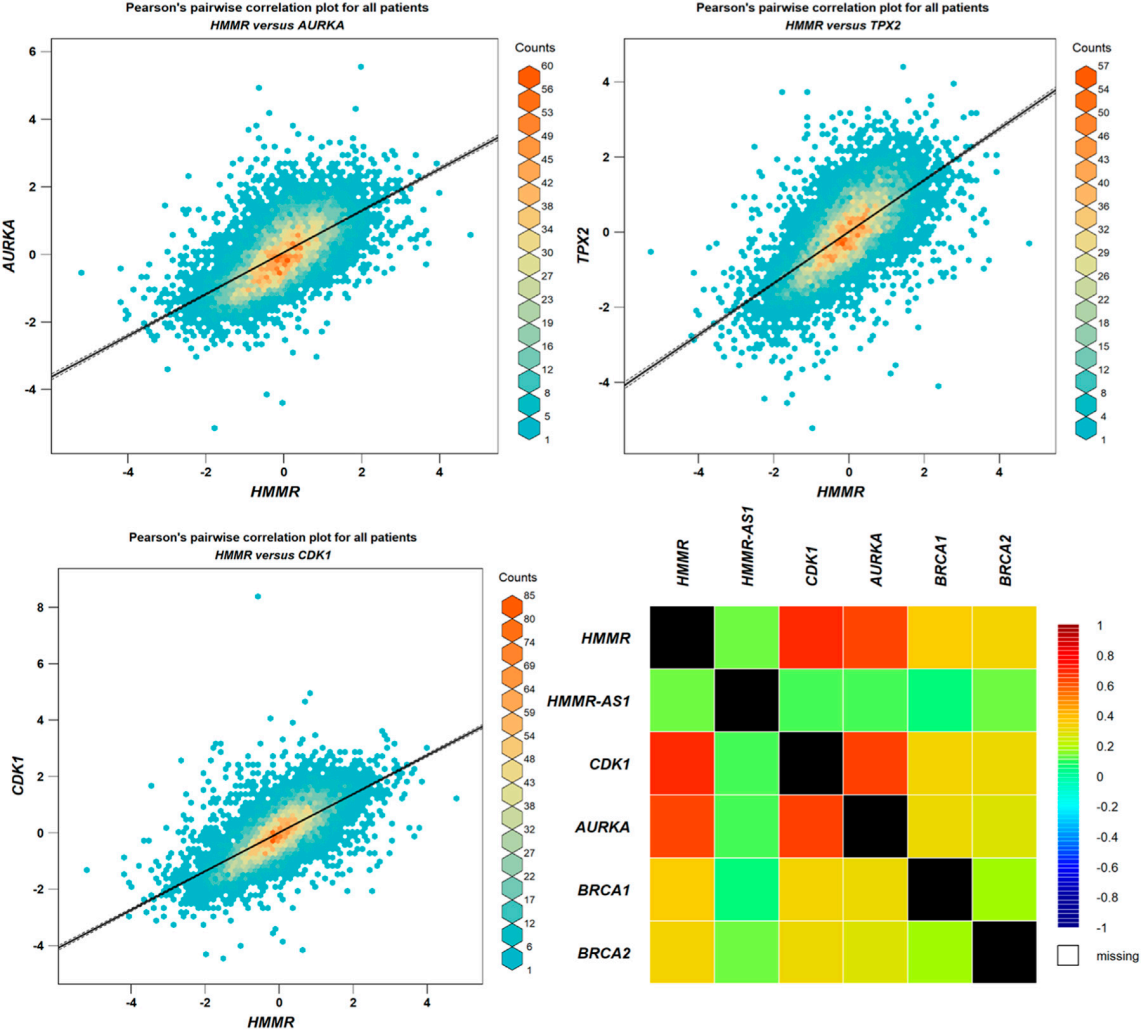
Analysis of correlation between HMMR and AURKA, TPX2, and CDK1 through plots obtained from bc-GeneExMiner.

**FIGURE 8:**
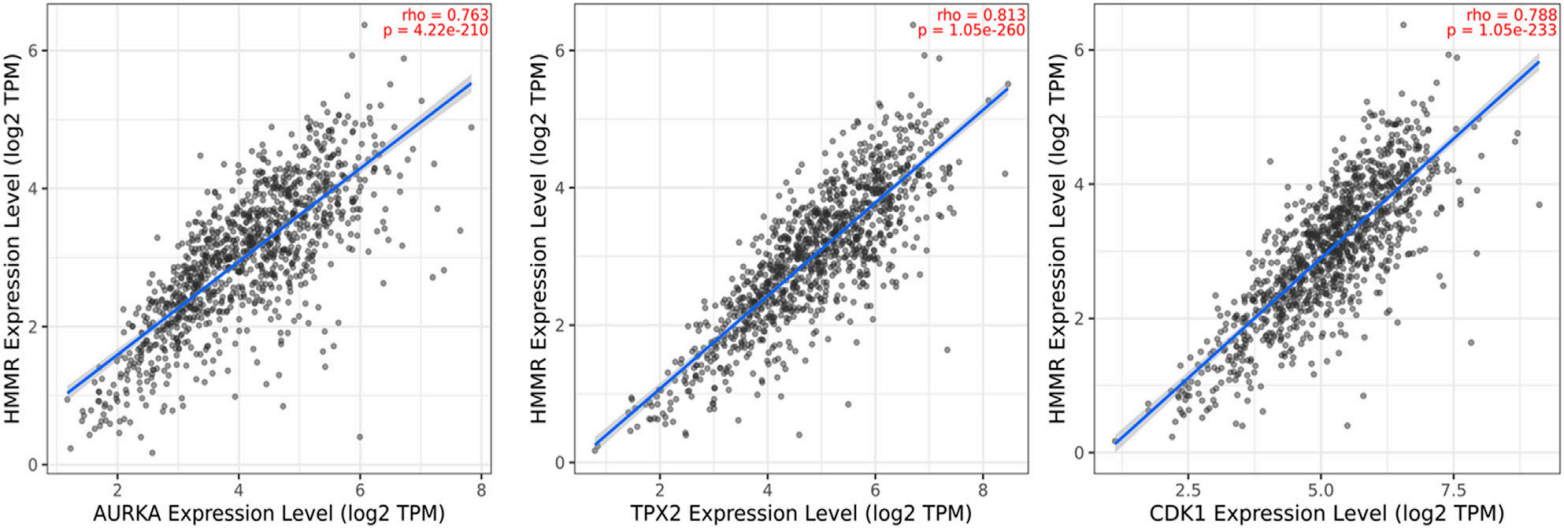
Gene correlation analysis through a linear regression graph between HMMR and AURKA, TPX2, and CDK1 across breast cancer where n = 1,100 (n: no. of patients) obtained from the TIMER 2.0. graphs show the highest correlation between HMMR and AURKA, followed by TPX2 and CDK1.

The data obtained from PPI interactions (Figure 5) and Gene Ontology (Figure 6) revealed certain potential target genes, such as *AURKA*, *TPX2*, and *CDK1*, which could be analyzed to check the correlation between HMMR and the above-mentioned genes. The heat map and Pearson’s pairwise correlation plot obtained from bc-GenExMiner indicated a high correlation between HMMR and the target genes (Figure 7). Figure 8 depicts the gene correlation analysis through a linear regression graph between HMMR and AURKA, TPX2, and CDK1 across breast cancer obtained from TIMER 2.0. The graphs show the highest correlation between HMMR and AURKA, followed by TPX2 and CDK1.

### 3.5 Modeling of protein and validation

**FIGURE 9:**
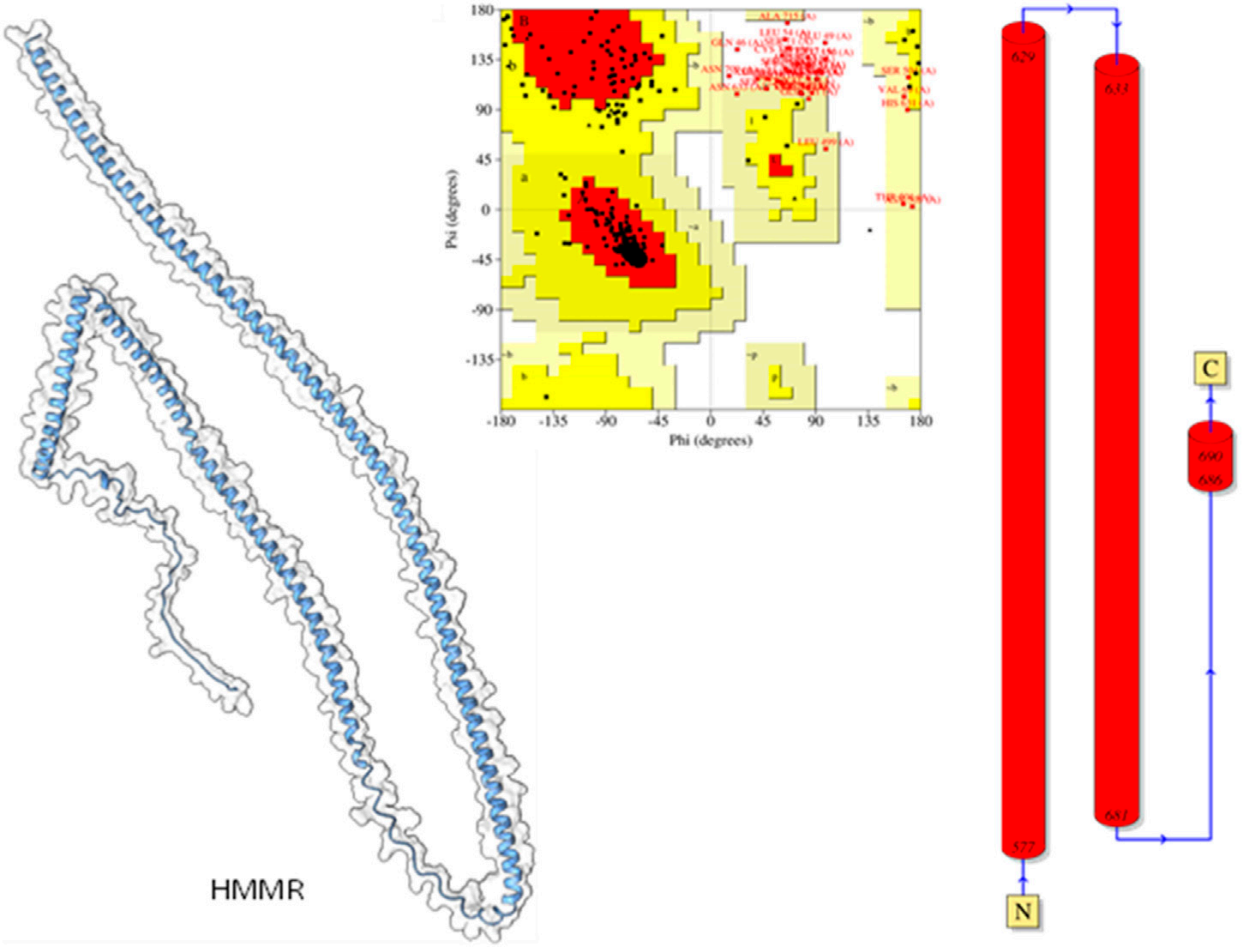
Modeled HHMR protein is in ghostly white cartoon representation; central panel, Ramachandran’s plot; right panel, 2D plot of the helix domains.

Modeled proteins using AlphaFold2 are displayed in Figure 9. Protein HMMR exhibited two longitudinally arranged α-helices, the C-terminal domain exhibited a small turn, and the rest conformed to extended loops at both N and C-terminals (Figure 9). The residual positions in the formation of the secondary structure were confirmed using Ramachandran’s plot (Figure 9). Residues are the most favored region, which was 88.9%, the additional allowed region was 6.25%, and the generously allowed region was 5.2%. No residues were seen at the disallowed region. Therefore, it could be suggested that the predicted model is well-validated and considered for further studies.

**FIGURE 10:**
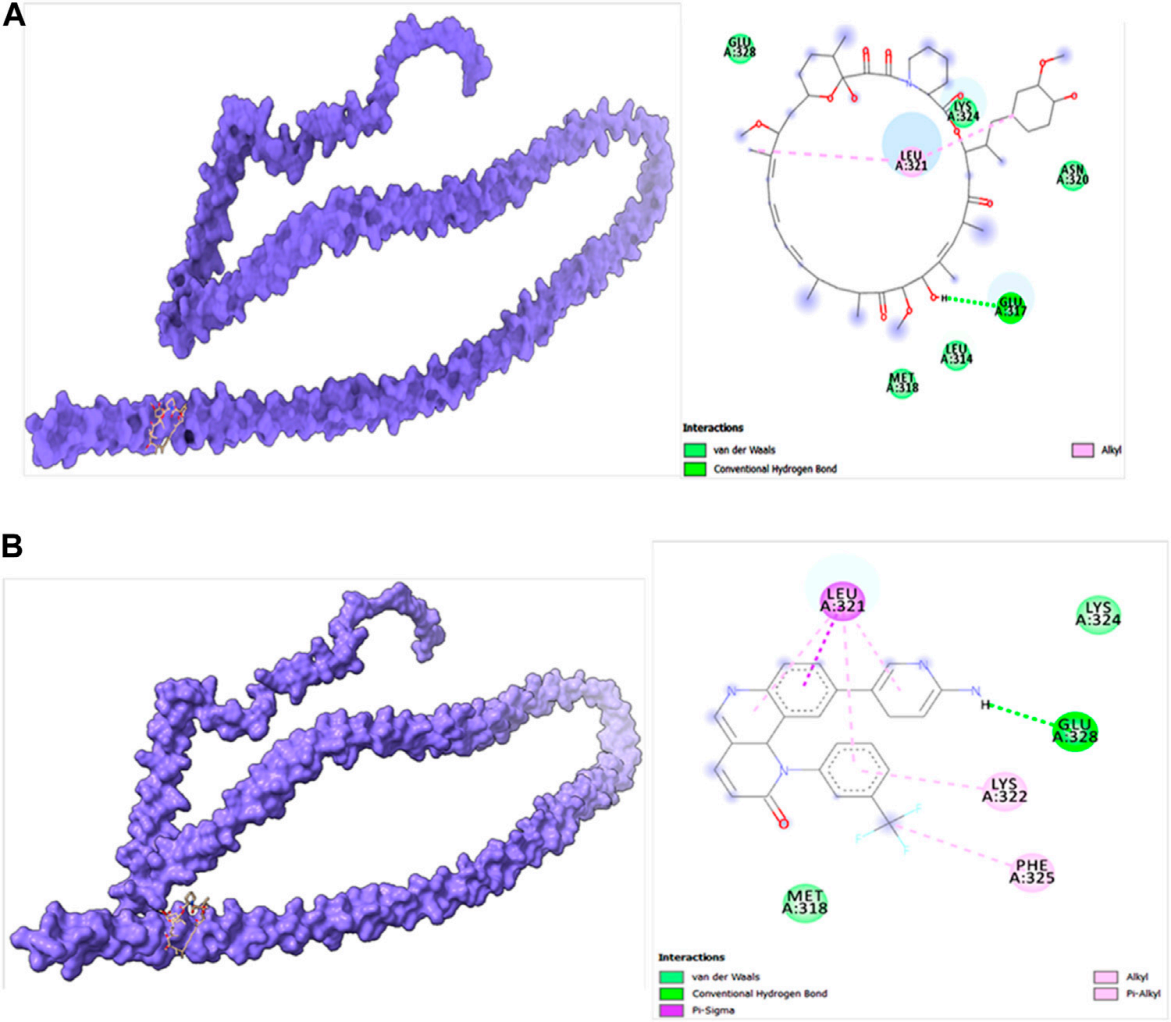
**(A)** Modeled HMMR docked with rapamycin. **(B)** Modeled HMMR docked with Torin2 exhibiting the frequency of populations at the 2.0 tolerance level. Surface view of proteins are exhibiting a deep core of binding pocket accommodating the ligands, 2D interaction plot of ligands binding pocket in the respective proteins.

**FIGURE 11:**
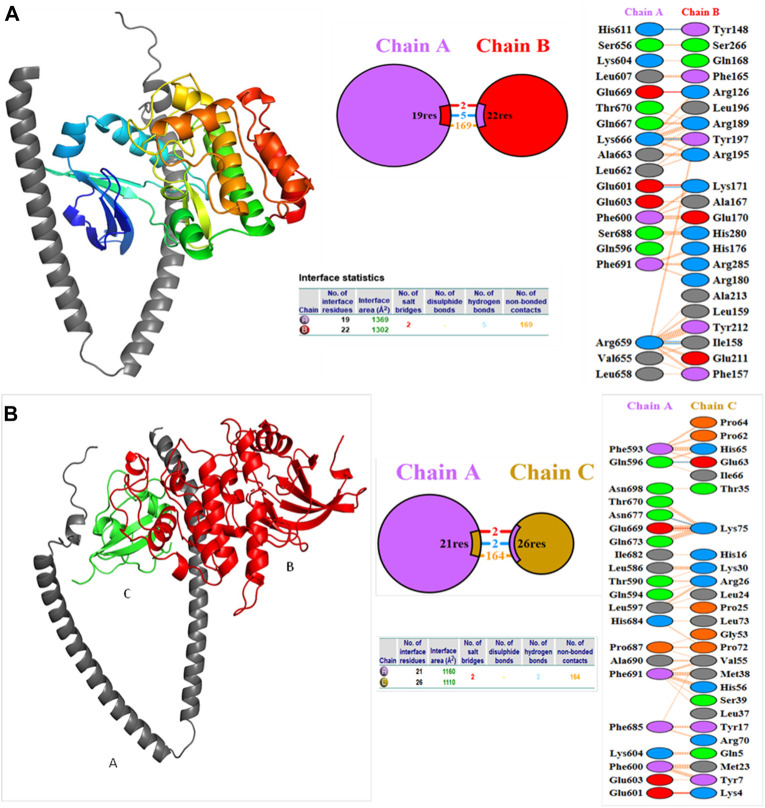
Structures of protein–protein docking best pose between **(A)** HMMR + AURKA and **(B)** HMMR + CDK1. The middle panel shows the interacting residue numbers where chain A is for HMMR and explains the number of residual interactions between HMMR and respective proteins, and the left panel shows the interacting residues.

Molecular docking studies were carried out to decipher the binding aspects of target HMMR with ligands such as rapamycin and Torin2. The images of molecular surfaces, docked complexes, and two-dimensional and three-dimensional interactive plots for targeting HMMR with ligands such as rapamycin and Torin2 are shown in Figures 10A and B. HMMR and rapamycin showed a considerable binding affinity of ΔG −5.4 kcal/mol. The residue Glu317 at the binding cavity was involved in conventional hydrogen bonding, while other residues of the cavity are involved in weak van der Waals interactions with rapamycin (Figure 10A). HMMR with the ligand Torin2 exhibited a binding energy of ΔG −5.9 kcal/mol. Here, the residue Glu328 was involved in conventional hydrogen bonding, while Phe325, Lys322, and Leu321 were involved in alkyl and pi–alkyl interactions. Residue Leu321 was also involved in the pi–sigma interaction (Figure 10B). No other significant interactions are observed. Protein–protein docking between HMMR + AURKA and HMMR + CDK1 is shown in Figure 11. The binding energy score calculated from the HDOCK server for HMMR + AURKA is −738.7, forming 169 non-bonded interactions (Figure 11), a couple of salt bridges, and five hydrogen bonds. Meanwhile, HMMR + CDK1 exhibited significant binding and a much lower dock score with the lowest energy of −515.9. Overall, 164 non-bonded interactions took place where two hydrogen bonds and a couple of salt bridges were formed (Figure 11). Therefore, from PPI docking, it could be suggested that HMMR had a higher interaction with AURKA compared to CDK1.

### 3.6 Molecular dynamics simulation

**FIGURE 12:**
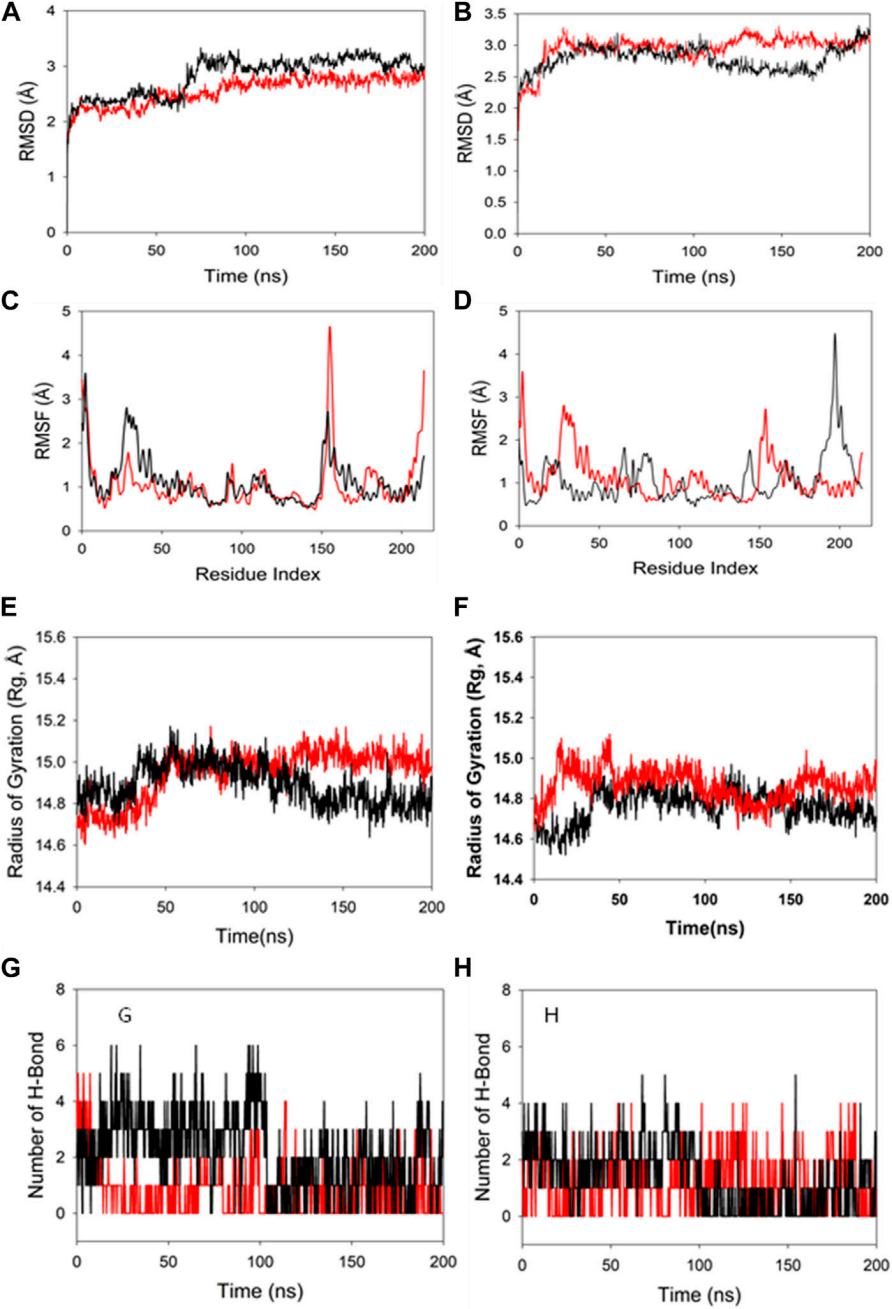
MD simulation analysis of 200-ns trajectories of **(A)** Cα backbone RMSD of HMMR + Torin2 (red), HMMR + rapamycin (black); **(B)** Cα backbone RMSD of HMMR + AURKA (red) and HMMR + CDK1 (black); **(C)** Cα backbone of HMMR + Torin2 (red) and HMMR + rapamycin (black); **(D)** Cα backbone RMSF of HMMR + AURKA (red) and HMMR + CDK1 (black). MD simulation analysis of 200-ns trajectories of **(E)** Cα backbone radius of gyration (Rg) of HMMR + Torin2 (red) and HMMR + rapamycin (black). **(F)** radius of gyration (Rg) of the Cα backbone of HMMR + AURKA (red) and HMMR + CDK1 (black). **(G)** Formation of hydrogen bonds in HMMR + Torin2 (red) and HMMR + rapamycin (black). **(H)** Formation of hydrogen bonds in HMMR + AURKA (red) and HMMR + CDK1 (black).

Molecular dynamics and simulation was carried out to assess the stability and convergence of HMMR in the presence of the drugs rapamycin and Torin2. The 200-ns simulations revealed consistent conformations, as evidenced by the comparison of root-mean-square deviation values. Specifically, the Cα-backbone RMSD of HMMR in complex with Torin2 displayed a deviation of 2.8 Å, while the protein that was bound to rapamycin exhibited a similar deviation of 2.8 Å (refer to Figure 12A). Importantly, all RMSD values remained below the acceptable threshold of 3 Å. The observed stability in the RMSD plots throughout the simulation indicates robust convergence and the maintenance of stable conformations. Hence, it can be proposed that pharmaceuticals attached to HMMR exhibit considerable stability within the complex due to the heightened affinity of the ligand. Similarly, HMMR, when bound to the AURKA protein, demonstrated an RMSD value of 2.91 Å, while with the Cdk1-bound protein, the RMSD was 3.0 Å (see Figure 12B). The root-mean-square fluctuation (RMSF) plot displayed minor peaks in fluctuation for the HMMR protein with Torin2, with the exception of notable spikes at residues 150–155, possibly indicating increased flexibility in these residues (Figure 12C). In the case of HMMR with rapamycin, fluctuations occurred at positions 30–45 and 150–175 (refer to Figure 12C). The HMMR bound to AURKA protein exhibited negligible fluctuations, suggesting a rigid protein conformation during ligand binding (Figure 12D). Conversely, the Cdk1-bound protein displayed residual fluctuations at residues 180–230 (Figure 12D). Most residues exhibited low fluctuations throughout the entire 200-ns simulation (Figures 12C and D), signifying stable amino acid conformations during the simulation period. Thus, the root-mean-square fluctuation plot suggests that the protein structure remains rigid during simulation of the conformations that are ligand-bound.

**FIGURE 13:**
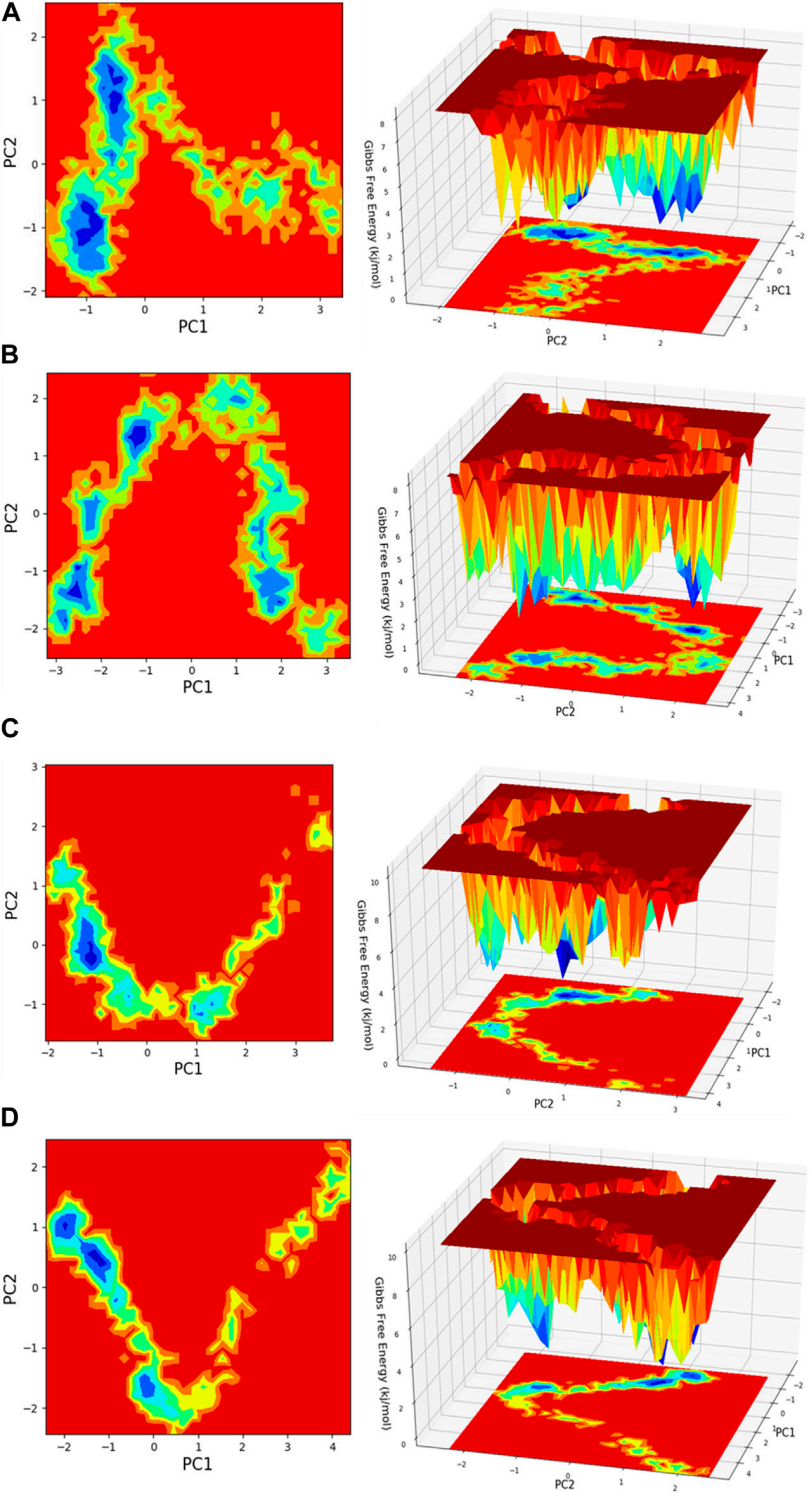
Free energy landscape (FEL) is depicted concerning principal components (PCs) for conformational scrutiny, where the left panel displays a 2D FEL along with clusters of frames. The structures at the midpoint are presented with a time scale, where the right panel showcases the well of global minima in a 3D representation. **(A)** HMMR + Torin2, **(B)** HMMR + rapamycin, **(C)** HMMR + AURKA, and **(D)** HMMR + CDK1.

The radius of gyration (Rg) serves as an indicator of the compactness of proteins. In this investigation, the Cα-backbone of HMMR bound to Torin2 consistently displayed an Rg value ranging from 14.8 to 15.0 Å (Figure 12E). In contrast, a distinct pattern was observed for the rapamycin-bound protein, with Rg values ranging from 14.8 to 14.7 Å (Figure 12E). The Rg values for HMMR bound to AURKA remained stable, ranging from 14.8 to 14.9 Å, and for HMMR bound to Cdk1, they fluctuated between 14.7 and 14.76 Å (Figure 12F). A significantly stable gyration (Rg) suggests a greatly compact orientation of the protein in the ligand-bound state. A significant interaction and stability of the complex are indicated by the number of hydrogen bonds between the protein and the ligand. The hydrogen bond analysis between HMMR and Torin2 revealed a notable count of two bonds, and with rapamycin, three hydrogen bonds were observed (Figure 13G). Similarly, between AURKA and HMMR, two hydrogen bonds were identified, and with CDK1, there were two hydrogen bonds observed, persisting throughout the entire 200-ns simulation period (Figure 12H).

### 3.7 Molecular mechanics generalized Born surface area

The binding free energy and additional contributing energy in the form of MM-GBSA were determined for each, HMMR + rapamycin and HMMR + Torin2. The binding free energy and additional contributing energy in the form of molecular mechanics generalized Born surface area were calculated for each of the two molecules, HMMR + rapamycin and HMMR + Torin2, using the molecular dynamics simulation trajectory. The findings (Table 1) indicated that ΔG_bind_Coulomb, ΔG_bind_vdW, and ΔG_bind_Lipo contributed the most to ΔG_bind_ in the stability of the simulated complexes, but ΔG_bind_Covalent and ΔG_bind_SolvGB contributed to the instability of the corresponding complexes. The binding free energies of the HMMR + rapamycin and HMMR + Torin2 complexes were considerably higher. These findings confirmed the strength of HMMR-containing rapamycin and Torin2 molecules. They also showed that these molecules could form stable protein–ligand complexes and were successful in binding to the specified protein.

**TABLE 1:** Binding free energy components for the HMMR + Torin2 and HMMR + rapamycin complex calculated by molecular mechanics generalized Born surface area.

Energy (kcal/mol)	HMMR + Torin2	HMMR + rapamycin
ΔG_bind_	−28.53 ± 4.1	−26.15 ± 1.13
ΔG_bind_Lipo	−19.83 ± 2.3	−13.43 ± 1.6
ΔG_bind_vdW	−12.68 ± 2.17	−14.160 ± 3.0
ΔG_bind_Coulomb	−2.14 ± 1.01	−6.22 ± 0.99
ΔG_bind_H_bond_	−0.06 ± 0.01	−0.62 ± 0.16
ΔG_bind_SolvGB	13.65 ± 2.27	21.2 ± 1.7
ΔG_bind_Covalent	0.85 ± 0.5	2.66 ± 1.12

#### 3.7.1 Principal component analysis and free energy landscape

Principal component analysis (PCA) is conducted on the MD simulation trajectories of proteins to interpret the randomly selected, statistically significant conformations (overall movement) of the atoms within the amino acid residues sampled throughout the trajectory, as illustrated in the figure. The movement of internal coordinates in three-dimensional space over a temporal duration of 100 ns was captured in a covariance matrix. The directional motion of each trajectory is explained as orthogonal collections or eigenvectors. During the trajectory, PCA highlights the statistically notable conformations. It allows for the identification of principal movements within the trajectory and the essential motions necessary for conformational alterations. The analysis involved examining two separate aspects, namely, the distance between Cα-atoms (PC1) and the dihedral angles Φ and Ψ (PC2) on 2D planes, as illustrated in Figure 13. This assessment allowed us to identify the primary movements occurring throughout the trajectory, including crucial reaction coordinates. Within the HMMR + Torin2 complex, several local minima were observed, each containing a significant number of frames. The clusters of local minima highlight significant transition barriers when comparing the principal components (see Figure 13A). In the case of HMMR + rapamycin, four to five clusters of local minima were observed. The presence of the drug-bound state suppresses protein activity and raises energy barriers. Frames within each specific cluster of local minima exhibit correlated motion individually, and the eigenvalues are recorded along both PC1 and PC2 axes (refer to Figure 13B). In the case of the AURKA protein complexed with HMMR protein, a solitary prominent cluster was observed within the local minima, indicating a deep energy well with fewer transition barriers, owing to reduced protein inhibition (see Figure 13C). For HMMR + CDK1, three distinct clusters of local minima islands were identified (see Figure 13D). These discrete clusters of energy minima suggest significant energy barriers due to drug inhibition, resulting in the protein undergoing conformational changes from its native folding pattern.

#### 3.7.2 Analysis of the secondary structure of proteins

**FIGURE 14:**
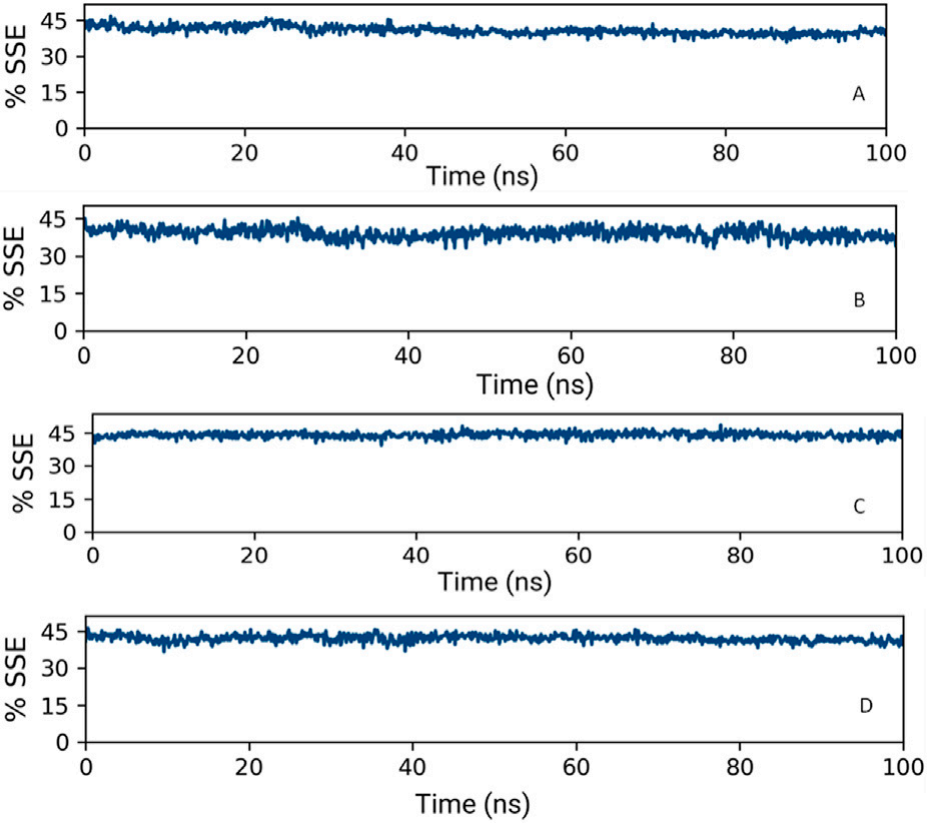
Secondary structure element percentage of **(A)** HMMR + Torin2, **(B)** HMMR + rapamycin, **(C)** HMMR + AURKA, and **(D)** HMMR + CDK1.

Upon analyzing the secondary structural elements (SSEs) crucial for the protein’s overall stability, it was noted that HMMR + Torin2 exhibited an approximate average of 41% SSE (Figure 14A), predominantly comprising helices rather than strands, whereas HMMR + rapamycin displayed an average of about 40% SSE (Figure 14B) in both its apo and ligand-bound states. During the 100-ns simulation, minimal alterations were noted in the snapshots of both the Torin2- and rapamycin-bound states of the HMMR protein structure. Conversely, in the HMMR + AURKA and HMMR + CDK1 configurations, the average secondary structural elements (SSEs) exhibited patterns akin to those observed in the Torin2-bound state, with the percentage of SSE remaining at 41% (Figures 14C and D). Throughout the simulation, the secondary structure of the HMMR protein remained relatively constant when bound to the hits, indicating the hits’ stability in the complex with both Torin2- and rapamycin-bound HMMR. Hence, the MD simulation indicates the stability of the complexes.

## 4 Discussion

As breast cancer is an issue of global health concern, it is very important for researchers to find potential avenues to target it and find potential therapeutic strategies. Therefore, in this regard, the versatile functions of HMMR, both extracellularly and intracellularly, underscore its significance in cancer biology (Sullivan et al., 2018). Its extracellular role is as a receptor for hyaluronic acid (HA)-regulated cell migration, a pivotal aspect in inflammation and wound healing (Aya and Stern, 2014). Intracellularly, HMMR’s association with the cell cycle, spindle assembly, and microtubules further solidifies its impact on cell growth and division (Nguyen et al., 2017). The fine-tuned regulation of HMMR in healthy tissues contrasts with its upregulation in proliferative tissues, particularly evident in various cancer types. This dysregulation contributes to invasiveness and metastasis, aligning with unfavorable prognosis in cancers such as colorectal, breast, and prostate carcinomas. The overexpression of HMMR in the mouse mammary epithelium influences the tumor microenvironment and cancer cell phenotype, leading to an increase in the genesis of Brca1-mutant tumors (Mateo et al., 2022).

The consistent overexpression of HMMR across various cancers, especially in breast cancer, underscores its potential as a significant biomarker. The UALCAN, TIMER 2.0, and GEPIA2 analyses reveal a substantial upregulation of HMMR in tumor samples, indicating its role in disease progression. The robustness of these findings across multiple databases enhances the credibility of HMMR as a clinically relevant marker. The AlphaFold2-based molecular modeling of HMMR provides a reliable structural framework for understanding its interactions. The subsequent docking studies with rapamycin and Torin2 offer insights into potential therapeutic interventions. The binding affinities and interaction patterns, especially the involvement of key residues, provide a rational basis for considering these compounds as inhibitors. The specificity of these interactions, as illustrated in the 2D and 3D plots, strengthens the argument for their candidacy as therapeutic agents. The 200-ns MD simulations provide a dynamic view of the stability of HMMR-ligand complexes and demonstrate the robustness of the structures, with minimal deviations and fluctuations. This stability, coupled with the maintenance of hydrogen bonds, underscores the potential of rapamycin and Torin2 to form enduring complexes with HMMR. Molecular mechanics generalized Born surface area-based binding free energy calculations offer quantitative measures of the energetics involved in HMMR–ligand interactions. Favorable binding energies for rapamycin and Torin2 suggest strong and stable binding. The breakdown of energy components highlights the significance of various forces contributing to the overall stability of the complexes.

Principal component analysis of molecular dynamics simulation trajectories provides valuable insights into the conformational dynamics of protein–ligand complexes. By analyzing the movement of atoms within amino acid residues, PCA identifies statistically significant conformations and principal movements essential for understanding protein dynamics (Shukla and Tripathi, 2020). In our study, PCA revealed distinct conformational landscapes for HMMR in complexes with different ligands. The HMMR + Torin2 complex exhibited multiple local minima, indicating significant transition barriers. Conversely, HMMR + rapamycin displayed fewer clusters of local minima, suggesting suppressed protein activity and elevated energy barriers due to the drug-bound state. This implies different modes of interaction and potential therapeutic implications for each complex. Notably, the HMMR + AURKA complex showed a prominent energy well with reduced transition barriers, indicative of a stable protein–ligand interaction. In contrast, HMMR + CDK1 exhibited multiple clusters of local minima, suggesting substantial energy barriers and conformational changes, potentially impacting protein function.

Analysis of SSEs further supported the stability of HMMR–ligand complexes. Both HMMR + Torin2 and HMMR + rapamycin maintained consistent secondary structure percentages throughout the simulation, indicating stable interactions between HMMR and the respective ligands. Similarly, HMMR + AURKA and HMMR + CDK1 configurations exhibited stable secondary structures, suggesting robust binding of HMMR with these proteins. Overall, our MD simulations demonstrate the stability of HMMR–ligand complexes and provide insights into their conformational dynamics. These findings contribute to our understanding of HMMR-mediated cellular processes and have implications for the development of targeted therapies in breast cancer treatment.

The proposed mechanism linking HMMR to the CDK1 complex provides insights into its potential role in accelerating the initiation of mitosis. This suggests a potential avenue for genetic modifications supporting tumor growth through increased CDK1/Cyclin B activity (Fulcher and Sapkota, 2020). The correlation between HMMR and key genes such as *AURKA*, *TPX2*, and *CDK1* highlights its involvement in crucial cellular processes. Importantly, these genes are the key regulators of the mTOR pathway, which is potentially known to be deregulated in various cancers. This provides a novel perspective on HMMR’s contribution to breast cancer progression. Notably, we exposed a plausible interaction between HMMR and AURKA, resulting in an elevation of AURKA protein levels. This augmentation subsequently triggered the mTORC2/AKT pathway, as proved in the case of prostate cancer (Miao et al., 2023). Therefore, we hypothesized that HMMR could be targeted through mTOR, which serves as a critical mediator in advancing the progression of breast cancer. The integration of the bioinformatics analyses and *in silico* studies leads to a compelling argument for the clinical relevance of HMMR and its potential as a therapeutic target. The proposed strategy of targeting the mTOR pathway with rapamycin and Torin2, based on the strong correlation with HMMR and associated genes, introduces a novel and promising avenue for breast cancer treatment. The proposed link between HMMR and the mTOR pathway signifies the potential therapeutic significance of mTOR inhibitors in breast cancer treatment. By disrupting this pathway, not only can we target HMMR but also modulate its associated genes, presenting a comprehensive approach to curb tumor growth.

Having said that, despite indications that increased levels of HMMR expression serve as predictive elements for breast cancer patients, all the data examined in our study originated from bioinformatics databases and computational studies. Furthermore, owing to its rare occurrence, there was a deficiency in publicly available data, potentially leading to statistical imprecision. To address this issue, additional datasets containing larger cohorts need to be incorporated. Hence, further inquiries are warranted to ascertain whether HMMR might be harnessed as diagnostic indicators or therapeutic targets in breast cancer, and therefore, it is crucial to acknowledge that further experimental validations, preclinical studies, and clinical trials are necessary to translate these findings into practical therapeutic applications. This study not only contributes to the understanding of HMMR’s role in cancer but also suggests novel therapeutic strategies. The outcomes suggest that HMMR may function as a critical oncogenic controller and a promising target for treating breast cancer.

## 5 Conclusion and future perspective

In conclusion, our study underscores the pivotal role of HMMR in cancer progression, providing a foundation for targeted therapeutic interventions. By unraveling the intricate networks involving HMMR and its correlation with key cellular processes, we pave the way for innovative strategies aimed at disrupting cancer growth and improving patient outcomes. The detailed structural insights and dynamic behaviors obtained from molecular modeling and dynamics simulations contribute to the overall understanding of HMMR as a potential therapeutic target in breast cancer. The findings not only highlight the clinical implications of HMMR but also propose a targeted therapeutic approach with specific inhibitors. Further research in this direction holds the potential to translate these findings into effective clinical applications for breast cancer and beyond. Nevertheless, an in-depth investigation and *in vitro* and *in vivo* studies for the validation of the link between HMMR and the mTOR pathway are significant to elucidate the precise function and mechanism of HMMR in breast cancer progression, which we will try to elucidate in our further studies.

## Data Availability

The original contributions presented in the study are included in the article/Supplementary material; further inquiries can be directed to the corresponding author.

## Abbreviations

HMMR: hyaluronan-mediated motility receptor, BC: breast cancer, AURKA: aurora kinase, TPX2: targeting protein for Xklp2, CDK1: cyclin-dependent kinase 1, mTOR: mammalian target of rapamycin, KEGG: Kyoto Encyclopedia of Genes and Genome, GO: Gene Ontology, OMIM: Online Mendelian Inheritance in Man, PPI: protein–protein interaction.
