# Erratum: Exploring HMMR as a therapeutic frontier in breast cancer treatment, its interaction with various cell cycle genes, and targeting its overexpression through specific inhibitors

**DOI:** 10.3389/fphar.2024.1443537

**Published:** 2024-06-21

**Authors:** Frontiers Production Office

**Affiliations:** Frontiers Media SA, Lausanne, Switzerland

**Keywords:** HMMR, breast cancer, biomarkers, Cdk1, AURKA, Tpx2, mTOR, MD simulation

Due to a production error, there was an error in **Affiliation 5**. Instead of “Respiratory Care Department, College of Applied Sciences Almaarefa University, Abha, Saudi Arabia,” it should read “Respiratory Care Department, College of Applied Sciences Almaarefa University, Diriya, Riyadh, Saudi Arabia.”

The publisher apologizes for this mistake. The original version of this article has been updated.

